# The effect of gender, piloting and segmental analysis on T1 quantification in normal individuals using MOLLI

**DOI:** 10.1186/1532-429X-16-S1-P66

**Published:** 2014-01-16

**Authors:** Ausami Abbas, Alison M Fletcher, Stephen Harden, Peter J Weale, Charles Peebles, James Shambrook, Andrew S Flett

**Affiliations:** 1 Cardiothoracic Radiology, University Hospital Southampton, Southampton, UK; 2 Cardiology, University Hospital Southampton, Southampton, UK; 3 Siemens PLC Healthcare, Frimley, UK

## Background

T1 mapping is a rapidly evolving field and is emerging as a novel quantitative tissue characterisation technique with potential applications in fibrosis, oedema, fat, iron and other patho-physiology. The impact of gender, segmental analysis and image piloting is not yet fully explored and has the potential to complicate the interpretation of observed differences.

## Methods

9 healthy volunteers and 7 patients with no evidence/history of disease were scanned on a Siemens Avanto 1.5T scanner and a 32 channel coil using the Siemens modified look locker inversion recovery (MOLLI) WIP version 448B (5:3:3 acquisition with inline motion correction and T1 map generation). All subjects had a 4 chamber, basal and mid ventricular short axis acquisition performed. In all slices, a single, slender ROI was placed in the mid wall of the left ventricular septum with meticulous attention to avoiding partial volume of blood pool. In addition, the mid ventricular short axis slice was segmented (with aggressive endo and epicardial border pruning) into 6 AHA segments to allow regional T1 analysis.

## Results

**Figure 1:**
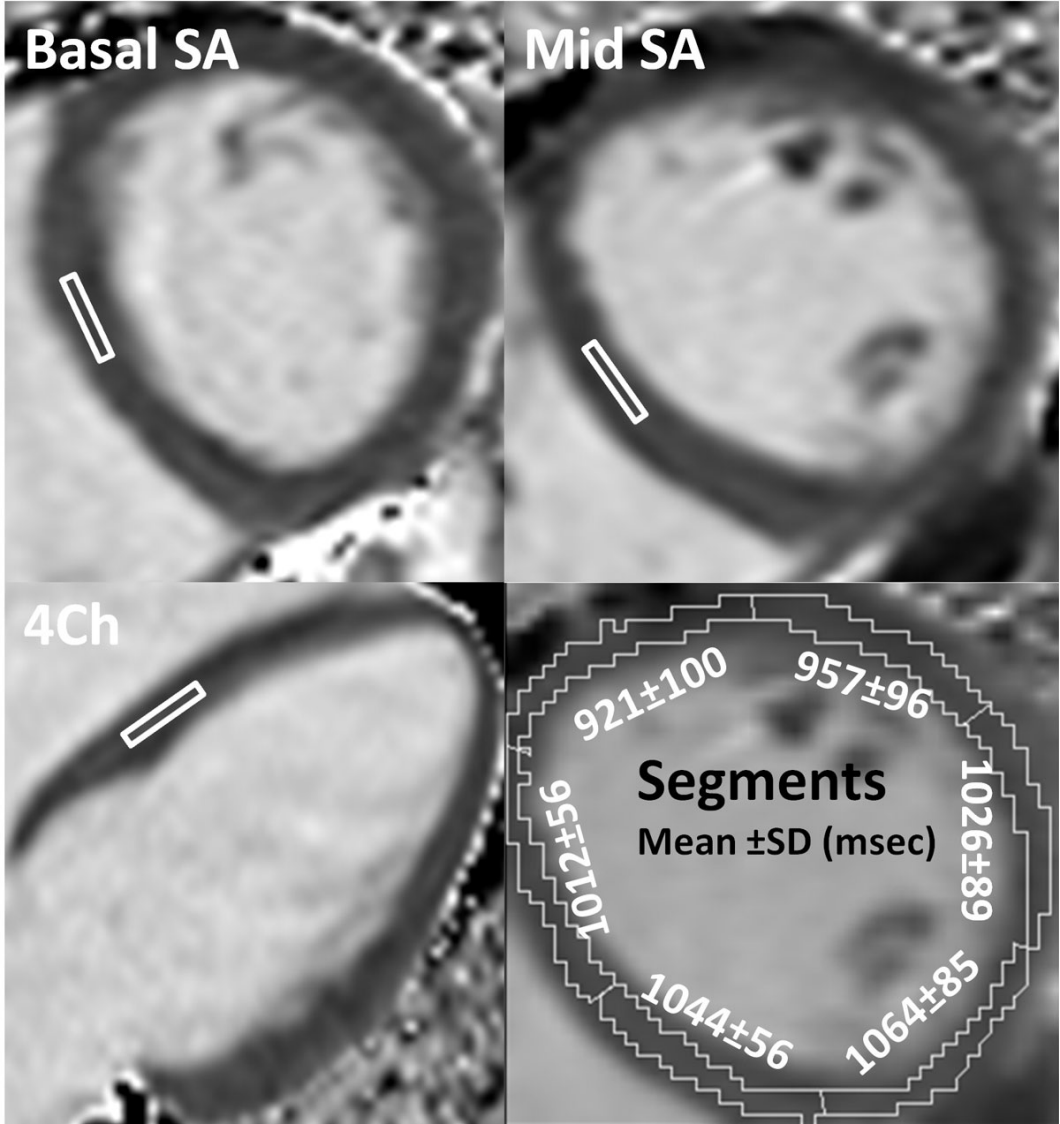
**Representative MOLLI's**. Segmented image shows mean T1's for each segment. SA = Short axis.

There were 9 females and 7 males, mean age 47. Mean T1 times are displayed in table 1 and representative images and ROIs in Figure 1. On the mid ventricular short axis and 4 chamber slices the mean myocardial T1 was significantly higher in females than males, p = 0.003. There was no difference in blood T1 between males and females. There was a non significant trend to higher T1 in the 4 chamber (1014 ± 42 ms) than in the mid short axis slice (999 ± 42 ms), p = 0.06. There was no difference between T1 at the base vs. mid ventricle. ROI based septal T1 did not differ from global T1 (p = 0.6). Global (mean of all segments) T1 was not significantly different to any segmental T1. The anterior segment had significantly shorter T1 than all other segments (p range < 0.0001 - 0.02).

**Table 1:** Mean T1 time in msec

	Females	Males	P(gender)	All
T1 myocardial Mid SA	1023 +/- 29	969 +/- 36	0.003*	1000 +/- 41

T1 myocardial Basal SA	1020 +/- 36	987 +/- 30	0.09	1006 +/- 36

T1 Myocardial 4-Chamber	1036 +/- 33	981 +/- 30	0.007*	1014 +/- 42

T1 Myocardial global	1039 +/- 54	959 +/- 31	0.003*	1004 +/- 61

T1 Blood Mid SA	1554 +/- 94	1564 +/- 70	0.6	1558 +/- 83

SA = Short Axis

## Conclusions

We present T1 times for MOLLI imaging in healthy individuals and demonstrate 1) significantly higher T1 in females than males 2) segment to segment T1 variation and 3) a trend to longer T1 in the 4 chamber compared to short axis. These results suggest that caution should be used when interpreting absolute T1 values. Differences may be observed by gender, slice orientation and segment. Whether these differences are due to true physiological (myocardial blood flow, difference in collagen quality/quantity, resident lipids) or artefactual (off resonance effects, motion correction effects, heart rate etc) differences is an area for further study.

## Funding

No external funding.

